# Data on the concentration-dependent score variations and the results of 2D correlation analysis in the measurements of H_2_SO_4_, HNO_3_, and H_3_PO_4_ samples

**DOI:** 10.1016/j.dib.2018.08.069

**Published:** 2018-09-01

**Authors:** Kyeol Chang, Hideyuki Shinzawa, Hoeil Chung

**Affiliations:** a Department of Chemistry and Research Institute for Convergence of Basic Sciences, Hanyang University, Haengdang-dong, Seongdong-gu, Seoul 133-791, Republic of Korea; b National Institute of Advanced Industrial Science and Technology (AIST), Nagoya 463-8560, Japan

## Abstract

Data presented here are related to the original paper “Concentration determination of inorganic acids that do not absorb near-infrared (NIR) radiation through recognizing perturbed NIR water bands by them and investigation of accuracy dependency on their acidities” published by same authors. Here, the concentration-dependent score variations and the results of 2D correlation analysis in the measurements of H_2_SO_4_, HNO_3_, and H_3_PO_4_ samples are included; while, the same analysis results obtained in the measurement of HCl samples are presented in the main manuscript. In addition, the correlation plots resulted in the measurements of HCl, H_2_SO_4_, HNO_3_, and H_3_PO_4_ samples are also separately shown.

**Specifications table**

**Table:** 

Subject area	Chemistry
More specific subject area	Analytical Chemistry, Near-infrared spectroscopy
Type of data	Figure
How data was acquired	NIR spectral data: ABB FT-NIR spectrometer (Quebec, Canada)
	PLS and 2D correlation analysis: Matlab R2016a
Data format	Text and Matlab formats
Experimental factors	Temperature and spectral resolution
Experimental features	Each sample was placed into a glass vial (inner diameter: 9.6 mm) and then into a metal vial holder with a temperature control precision of ± 0.1 °C. The temperature of all samples was maintained at 25.0 °C during collection of spectra.
Data source location	Seoul, Korea
Data accessibility	Ask to the authors for the NIR spectral data
Related research article	K. Chang, H. Shinzawa, H. Chung, Concentration determination of inorganic acids that do not absorb near-infrared (NIR) radiation through recognizing perturbed NIR water bands by them and investigation of accuracy dependency on their acidities, Microchemical Journal, in press

**Value of the data**

• Concentration-dependent score variations in the measurements of H_2_SO_4_, HNO_3_, and H_3_PO_4_ samples are compared with that of HCl samples.
• Also, 2D correlation analysis in the measurements of H_2_SO_4_, HNO_3_, and H_3_PO_4_ samples are also compared with that of HCl samples.
• The interactions between each inorganic acid and water molecules were dissimilar.
• Components with higher acidity, such as HCl, perturbed the water hydrogen bonding network more strongly.

## 1 Data

**Fig. 1:**
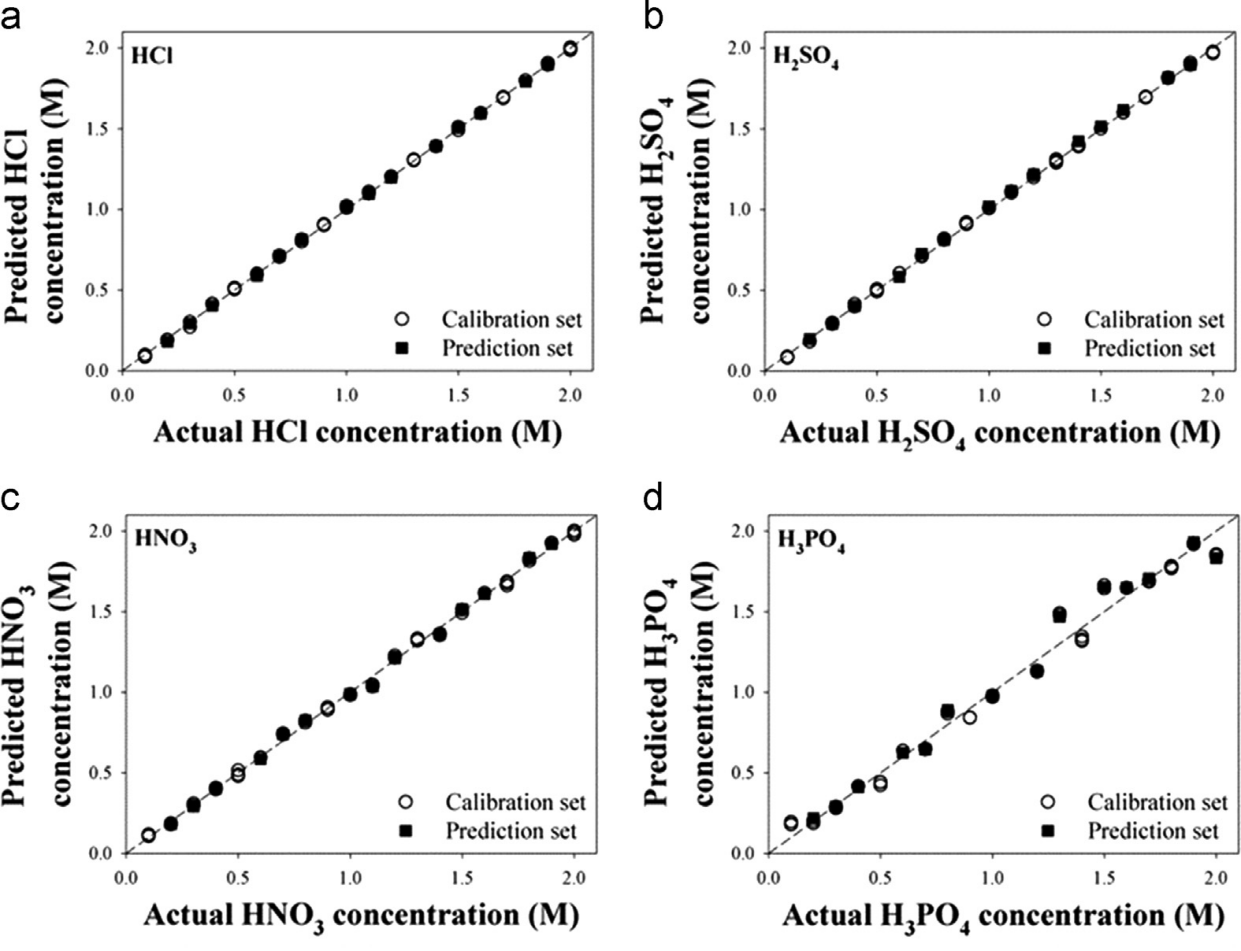
Correlation plots showing the actual vs. predicted concentrations for (a) HCl, (b) H_2_SO_4_, (c) HNO_3_, and (d) H_3_PO_4_.

**Fig. 2:**
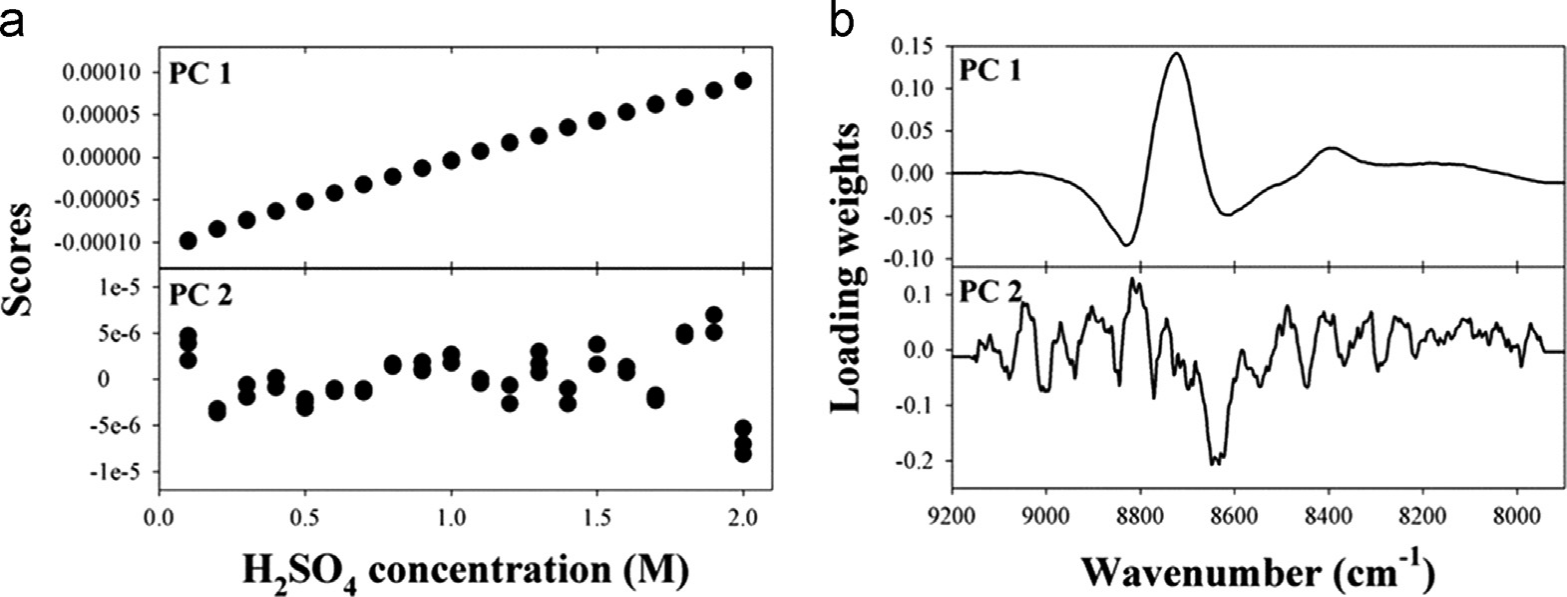
(a) First two scores of each sample and (b) corresponding loadings in the determination of H_2_SO_4_ concentration using PLS.

**Fig. 3:**
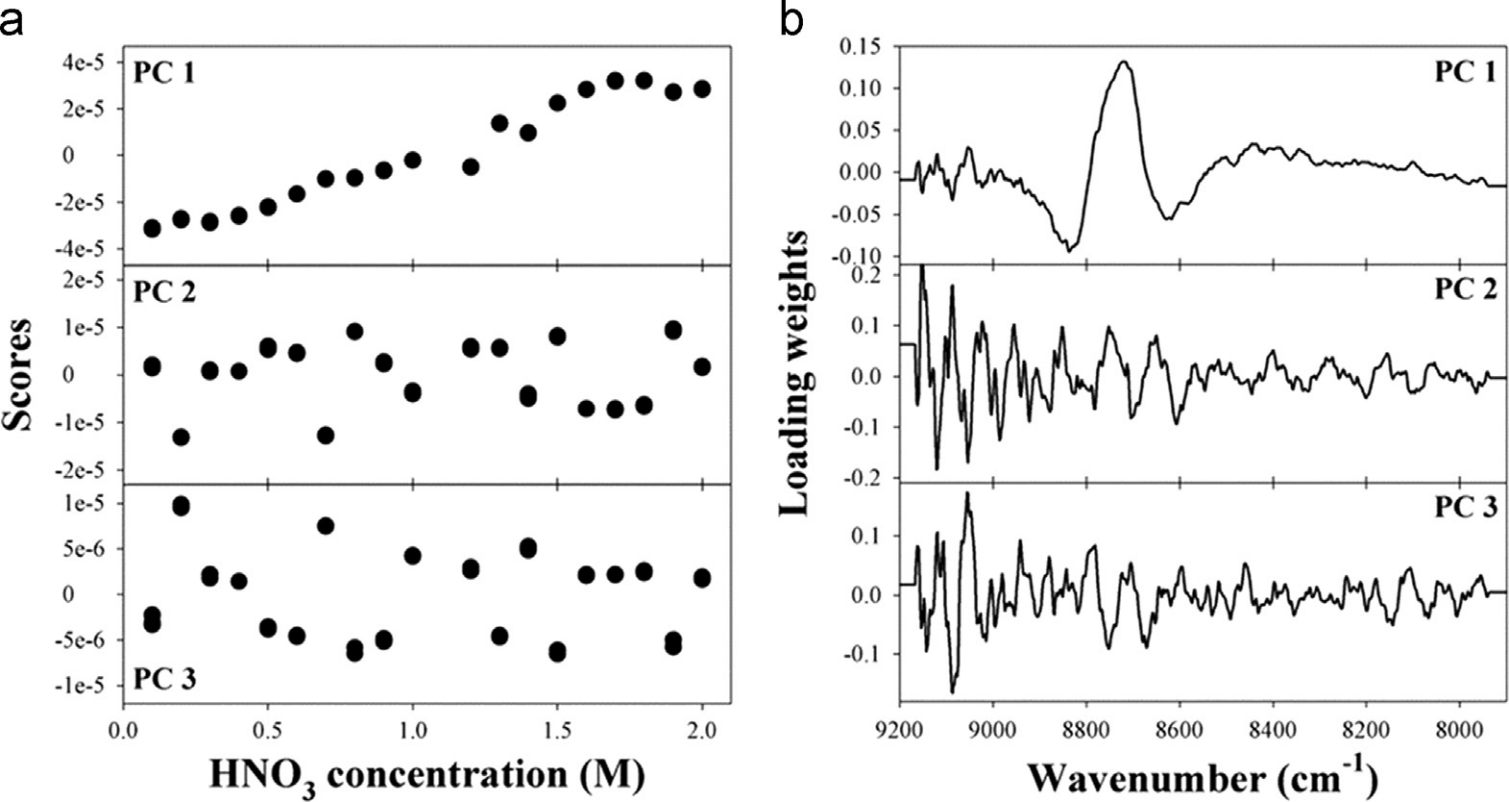
(a) First three scores of each sample and (b) corresponding loadings in the determination of HNO_3_ concentration using PLS.

**Fig. 4:**
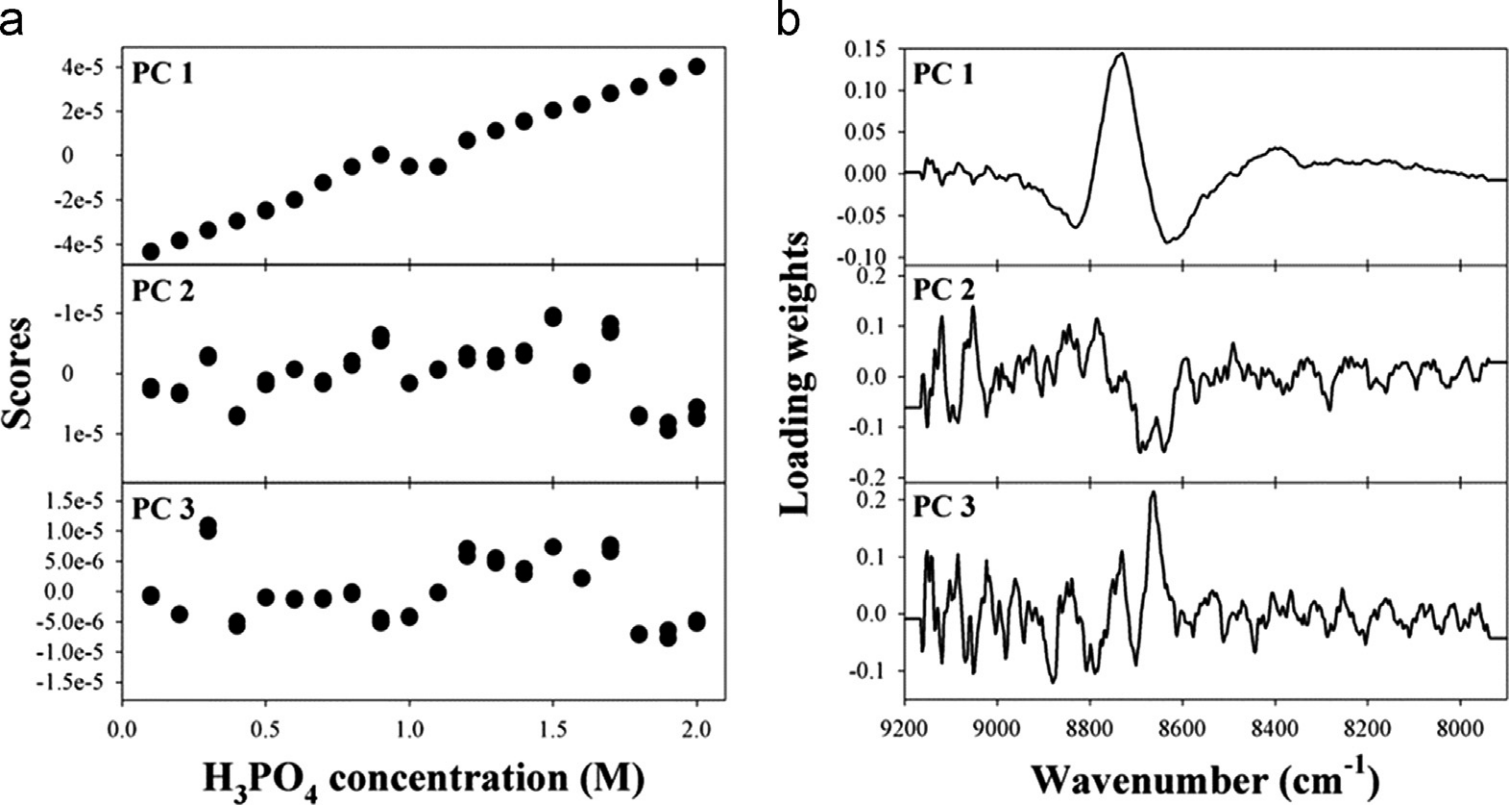
(a) First three scores of each sample and (b) the corresponding loadings in the determination of H_3_PO_4_ concentration using PLS.

**Fig. 5:**
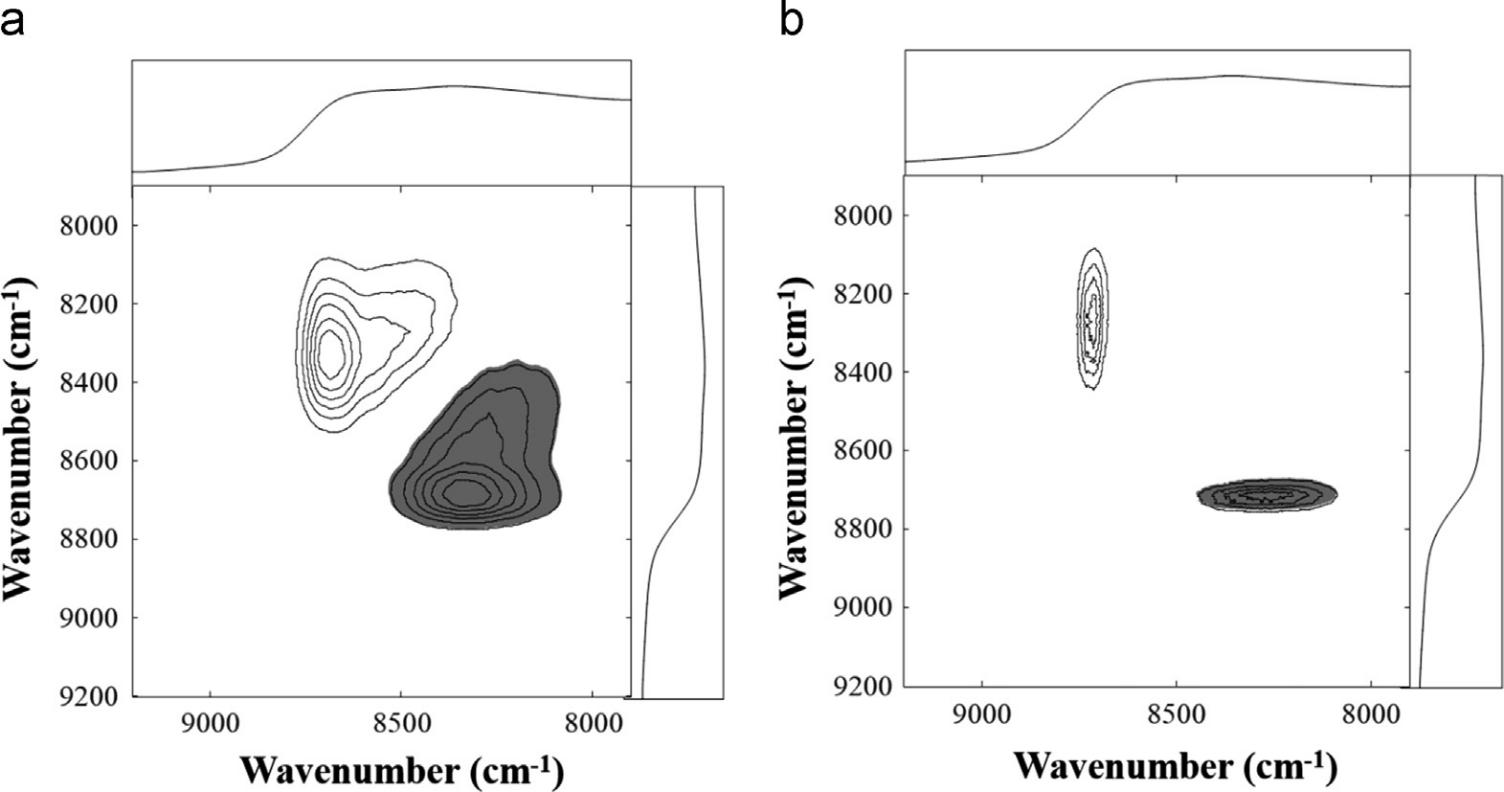
(a) Synchronous and (b) asynchronous correlation maps derived from the spectra of H_2_SO_4_ samples. Reference spectra are located at the top and sides of the plots.

**Fig. 6:**
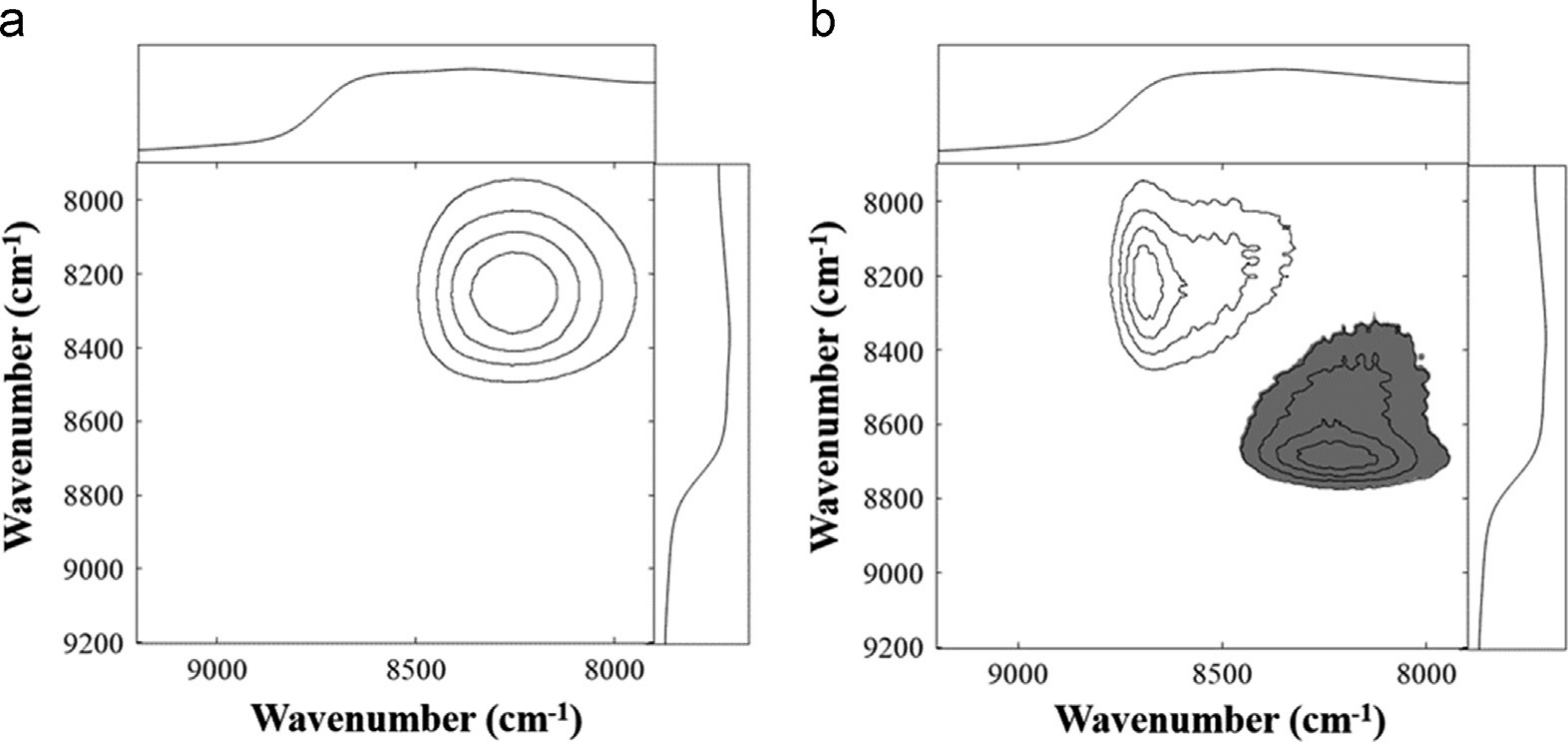
(a) Synchronous and (b) asynchronous correlation maps derived from the spectra of HNO_3_ samples. Reference spectra are located at the top and sides of the plots.

**Fig. 7:**
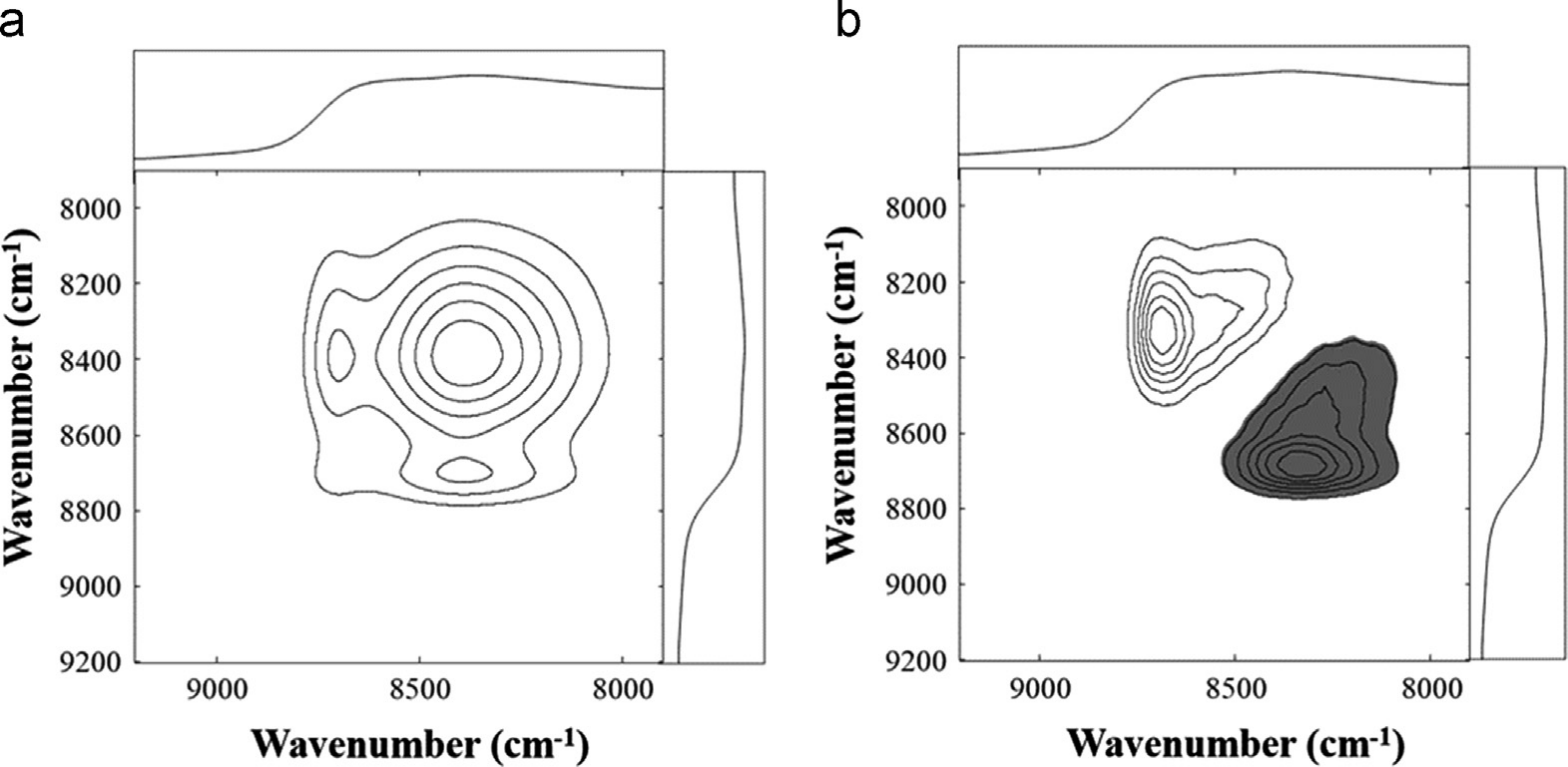
(a) Synchronous and (b) asynchronous correlation maps derived from the spectra of H_3_PO_4_ samples. Reference spectra are located at the tops and sides of the plots.

The dataset of this article provides supplementary information related to the original paper “Concentration determination of inorganic acids that do not absorb near-infrared (NIR) radiation through recognizing perturbed NIR water bands by them and investigation of accuracy dependency on their acidities”. The Fig. 1 describes concentration correlation plots of single-component samples. Fig. 2, Fig. 3, Fig. 4 show the concentration-dependent score variations and Fig. 5, Fig. 6, Fig. 7 show the results of 2D correlation analysis in the measurements of H_2_SO_4_, HNO_3_, and H_3_PO_4_ samples.

## 2 Experimental design, materials, and methods

### 2.1 Concentration correlation plots

Fig. 1 describes correlation plots showing the actual vs. predicted concentrations in the measurements of (a) HCl, (b) H_2_SO_4_, (c) HNO_3_, and (d) H_3_PO_4_ samples as described in Table 1 (refer to the main manuscript).

### 2.2 Concentration-dependent score variations

Fig. 2, Fig. 3, Fig. 4 show the concentration-dependent score variations in the measurements of H_2_SO_4_, HNO_3_, and H_3_PO_4_ samples, which are quite similar to those for the measurement of HCl as shown in Fig. 4 (refer to the main manuscript).

### 2.3 2D correlation analysis

Fig. 5, Fig. 6, Fig. 7 show the synchronous and asynchronous correlation maps derived from the spectra of in the measurements of H_2_SO_4_, HNO_3_, and H_3_PO_4_ samples.

