# Supplementary material for: Data on the concentration-dependent score variations and the results of 2D correlation analysis in the measurements of H2SO4, HNO3, and H3PO4 samples
Source: Data Brief. 2018 Sep 1;20:1422–7. doi: 10.1016/j.dib.2018.08.069 (PMC6148708; doi:10.1016/j.dib.2018.08.069)
Supplement: Supplementary file 1 — Transparency document [file mmc1.docx]

**Conflict of Interest**

To the best of our knowledge, the named authors have no conflict of interest, financial or otherwise.
